# Dermatology

**Published:** 1897-02-20

**Authors:** 


### DERMATOLOGY.

(*Continued from page* 318.)

**Acne.**—Hallopeau,[15] in a clinical lecture upon acne, draws attention to the various functions of the sebaceous glands. In the first place they are the seat of an unceasing formation of new epithelium, for it has been shown that their orifices and those of the sweat glands are the principal focus of epidermic production. The change in the quantity of this production is, doubtless, the cause of the phenomena of acne cornea; they also form an excretory apparatus for fatty substances ingested, or formed in the organism; thus excessive production, or change in these substances, becomes a cause of changes in the glands; they also eliminate substances abnormally introduced into or produced in the organism, which explains the cause of various medicinal (iodine, bromine) and of the so-called ptomainic forms of acne. Importance is also attached to the influence of over-feeding, more particularly over-indulgence in starchy and fatty food and in alcohol. He gives a long description of acne vulgaris, and then points out the differences between this and the other forms. With regard to treatment, the most important applications are sulphur, ichthyol, naphthol, thiol, carbolic and salicylic acids, and mercurial preparations. The amount of such active principles in an ointment must be regulated according to the reaction of the individual, since in some they cause severe inflammation of the integument. A steam spray appplication of sulphurous mineral waters has frequently proved efficacious.

MacDonnell,[16] besides enumerating the ordinary causes of the disease, gives a list of eighteen or twenty others, showing that acne can be produced by almost anything, from indigestion to a state of self-consciousness. In all non-suppurating cases, a preparation containing 10 parts of beta-naphthol, 50 of sulphur, 20 of green soap, and 20 of vaseline is recommended; this is spread on the affected surface, allowed to remain for about three-quarters of an hour, and then wiped off; this application should be made every evening for five days. To prevent the return of the trouble a 5 per cent. resorcin soap is recommended.

Hutchinson[17] considers acne very hereditary, especially the rosaceous form. This form, though frequently due to intemperance, however, must not be ascribed to this in every case. He states that people with very thin skins do not have acne, since the disease is only possible in those who have large sebaceous follicles.

Ohmann-Durnesuil[18] divides acne resacea into three stages: The hyperæmic, the inflammatory, and the hypertrophic. Many cases, however, are observed in which the disease does not progress beyond the first or second stage, becoming chronic in that particular form. In the first stage seborrhœa co-exists, and this form is apt to be persistent in women, but not so in men. With regard to treatment, he recommends the following sulphur lotion: R. Calcis vivæ ½ an oz., sulphur sublimate 1 oz., aquæ 10 oz.; to be boiled down to 6 oz. and filtered, and then should be perfectly clear. It is to be applied at night. In the third stage he gives the name of rhinoplyma, and recommends the removal of the entire mass by the knife.

**Therapeutics.**—Hartzell,[19] in an article on the uses of resorcin in dermatology, remarks that, although the drug has been employed for fifteen or twenty years, it does not occupy the position in dermatological therapeutics that it deserves. In eczema it allays pruritus and diminishes hyperæmia and exudation, and hastens the formation of cornified epithelium. The best results are obtained when it is used in watery solutions, in the proportion of from 10 to 15 grains to an ounce of water. It is rarely advisable to use stronger solutions, and ointments are apt to be irritating; the addition of half to one per cent. of sodium chloride seems to increase the sedative effects of aqueous solutions. In moist eczema the addition of some insoluble powder, such as oxide of zinc or bismuth (especially the subgallate) enhances its usefulness. The following lotion is recommended: ℞. Resorcin 10 to 15 grains, bismuth subgallate 30 grains, glycerine 10 minims, lime water an ounce. M. He also considers resorcin of use in cases of psoriasis, where tar or chrysarobin are objectionable; an ointment containing 30 to 40 grains to the ounce can be employed. In seborrhœa resorcin is one of the most useful remedies, as it is odourless, colourless, and makes an agreeable application to the scalp. A small quantity of the following oil may be rubbed in every night, but it should not be used with very light hair, as it causes some discolouration: ℞. Resorcin 20 grains, alcohol 2 drachms, vaseline 6 drachms. Occasionally a resorcin ointment may be used in cases of acne, but it sometimes sets up severe dermititis when used on the face. In epithelioma it sometimes acts in a remarkable way in promoting cicatrisation of superficial growths on the face. Finally, he has found it useful in favus trichophytosis.

Tauzer[20] recommends a soap containing 7 per cent. of nicotine in the treatment of parasitic dermatoses, and of other pruritic affections. It should be used with caution in children, since it may cause vomiting and cardiac depression.

Daulos[21] calls attention to a preparation of arsenic called cacodylic acid, by the use of which a much larger dose of arsenic can be given than by any other arsenical preparation, since in spite of its solubility it appears to have little toxic effects.

Unna[22] proposes the following basis for applying drugs to the skin: It is a gelatine paste, which contains 2½ per cent. each of gelatine and tragacanth, and can be used with drugs which could not be used in gelatine alone.

Swift[23] records his experience of thyroid feeding in skin affections. Besides improvement in psoriasis and chronic eczema, he obtained a satisfactory result in two cases of long-standing acne, and a great amount of benefit in all the cases of ichthyosis and the allied conditions of xerodermia and sclerodermia; no improvement was observed in cases of alopecia areata, urticara, impetigo, and erythema. He commences by giving one five-grain tabloid daily, and gradually increases the dose.

Morgan Muren[24] gives his experience of thyol in the treatment of ulcers and other skin affections. He considers it infinitely superior to ichthyol, since it has none of its offensive odour, and accomplishes all and a little more than that drug can do. It is freely soluble in water, and is non-irritating; it gave excellent results in eczema and in tinea tonsurans. Several cases are quoted.

[15] Med. Week., Sep. 18, 1896, p. 445. [16] N. Y. Med. Rec., Aug. 21, 1896. [17] Clin. Journ., Sep. 30, 1896. [18] Tri. State Med. Journ., Sep,, 1896, p. 411 [19] Ther. Gaz., June 15, 1896, p. 363; N. Y. Med. Rec, June 27, 1896, p. 853. [20] Nour. Rem., March 24, 1896, p. 287. [21] Med. Week, June 20, 1896. [22] Lancet, August 8, 1896. [23] Amst. Med. Gaz., No. 2-8; B M. J., Oct. 3, 1896. [24] N. Y. Med. Journ., June 25, 1896, p. 842.

